# Adverse drug reactions in hospitalized Colombian children 

**Published:** 2016-09-30

**Authors:** Roxana de las Salas, Daniela Díaz-Agudelo, Francisco Javier Burgos-Flórez, Claudia Vaca, Dolores Vanessa Serrano-Meriño

**Affiliations:** 1 Grupo de Investigación en Enfermería, Departamento de Enfermería, Universidad del Norte, Barranquilla, Colombia; 3 Biomimetics Laboratory, Instituto de Biotecnología Universidad Nacional de Colombia, Bogota, Colombia; 3 Departamento de Farmacia, Universidad Nacional de Colombia, Bogotá, Colombia

**Keywords:** Drug-related side effects, adverse reactions, pharmacovigilance, child, drug monitoring, Pediatrics, Risk, Colombia

## Abstract

**Introduction:**

The occurrence of adverse drug reactions is an important issue due to the lack of drug safety data in children.

**Objective:**

To describe the Adverse Drug Reactions in inpatient children under 6 years of age in two general pediatrics wards located in Barranquilla, Colombia.

**Methods:**

A prospective cohort study based on intensive pharmacovigilance was conducted during six months in order to monitor the emergence of Adverse Drug Reactions in inpatients children under 6 years of age with at least one medication prescribed. The study was conducted in two pediatric wards of two hospitals located in Barranquilla, Colombia. Naranjo´s Algorithm was used to evaluate imputability, the modified Hartwig and Siegel assessment scale to establish severity and the Schumock and Thornton criteria to determine preventability.

**Results:**

Of a total of 772 monitored patients, 156 Adverse Drug Reactions were detected on 147 children. The cumulative incidence of Adverse Drug Reactions was 19.0% (147/772); the incidence density was 37.6 Adverse Drug Reactions per 1,000 patients-days (147/3,913). The frequency was higher in children under 2 years of age (12.7%). Emergence of Adverse Drug Reactions was higher in male patients (RR= 1.66; 95% CI= 1.22-2.22, *p*= 0.001) and in those who used systemic antibiotics (RR= 1.82; 95% CI= 1.17-2.82, *p*= 0.005).

**Conclusions:**

Adverse Drug Reactions are common among hospitalized children and represent an additional burden of morbidity and risk, particularly in those who used several medicines, including antibiotics.

## Abstract (es)

**Introducción:**

La aparición de reacciones adversas a medicamentos es un tópico importante debido a la escasa información sobre seguridad de medicamentos en niños.

**Objetivo:**

Describir las reacciones adversas a medicamentos en niños menores de 6 años de edad hospitalizados en dos servicios de pediatría general en Barranquilla, Colombia.

**Métodos:**

Estudio prospectivo de una cohorte de pacientes basado en farmacovigilancia intensiva, realizado durante seis meses para monitorizar la aparición de reacciones adversas a medicamentos en niños menores de 6 años de edad hospitalizados y con indicación de al menos un medicamento. El estudio fue conducido en dos servicios de pediatría general de dos hospitales en Barranquilla, Colombia. El algoritmo de Naranjo fue usado para evaluar imputabilidad, la escala modificada de Hartwig y Siegel para establecer severidad y los criterios de Schumock y Thornton para determinar evitabilidad.

**Resultados:**

En total se monitorizaron 772 pacientes. Se detectaron 156 reacciones adversas a medicamentos en 147 niños. La incidencia acumulada de las reacciones adversas a medicamentos fue 19.0% (147/772); la densidad de incidencia fue de 37.6 reacciones adversas a medicamentos por 1,000 pacientes-día (147/3,913). La frecuencia de reacciones adversas fue mayor en niños <2 años de edad (12.7%). La ocurrencia de reacciones adversas a medicamentos fue mayor en pacientes masculinos (RR= 1.66; IC 95% =1.22-2.22, *p*= 0.001) y en quienes usaron antibióticos sistémicos (RR= 1.82; IC 95% = 1.17-2.82, *p*= 0.005).

**Conclusiones:**

Las reacciones adversas a medicamentos son comunes en niños hospitalizados y representan una morbilidad adicional y mayor riesgo, particularmente en aquellos que usaron varios medicamentos, incluyendo antibióticos.

## Introduction

The troubles related to pharmacological research in children have generated lack of drug safety and efficacy information 1, 2. Despite the effort of National and International authorities to stimulate the notification of Adverse Drug Reactions (ADRs), under-reporting is still quite common 3. Therefore, Intensive Pharmacovigilance is considered an important issue in pediatric population, particularly because of its increased susceptibility to ADRs 4,5 and predisposing factors 6. Intensive pharmacovigilance is the systematic monitoring of the occurrence of adverse events resulting from drug use during the entire length of prescription 7. According to the WHO, an ADR is "a response which is noxious and unintended, and which occurs at doses normally used in humans for the prophylaxis, diagnosis, or therapy of disease, or for the modification of physiological function" 8.

The WHO Global Individual Case Safety Report (ICSR) database (VigiBase (r)) has reported ADRs rates of 7.7% (268,145) in children from 0 to 17 years of age 9. However, these reports did not specify the frequency of ADRs in children under 6 years of age and seemed to show underestimated rates, as other studies have reported higher incidences 10. In addition, younger ages (children from 1 month to 2 years old), male sex, prolonged and previous hospitalization, indication of antibiotics and a higher number of prescribed drugs are factors associated with a higher risk of ADRs 10,11. In the case of Colombia, no specific data about ADRs frequency and characteristics has been described for this population, which is considered a priority in the sustainable development goals. Thus, the aim of this study was to describe the Adverse Drug Reactions in inpatient children under 6 years of age in two general pediatrics wards located in Barranquilla, Colombia.

## Materials and Methods

### Study design and participants

A prospective cohort study based on intensive pharmacovigilance was conducted during 6 months from June to December 2013 in two general pediatrics wards of two Colombian teaching hospitals in Barranquilla (city of the Colombian Caribbean Coast). One hospital was public with 29-bed capacity pediatric units, two of which are used for isolated patients. The other hospital was private and included 20 bed capacity pediatric units, from which isolation beds are assigned on a need basis. Both institutions admit patients between 1 month and 17 years of age. 

We included pediatric patients <6 years of age without ADRs which were hospitalized at least 24 hours and had at least one prescribed medication. Patients who did not meet the last inclusion criteria, or those whose parents did not authorize participation, or were admitted only for taking diagnostic test or referred from other institutions, were excluded. In addition, we did not monitored side effects associated with the administration of intravenous solutions, contrast media, nutraceuticals and topical products (dermatological and ophthalmic). Thus, we only monitored those drugs which contained active ingredients used for the treatment of illness.

During the monitoring period, 777 patients were admitted in the general pediatrics wards, of which 5 were excluded for being released before completing 24 hours of hospitalization. For this reason, we included 772 pediatrics patients <6 years of age. 

### Data collection

Once the study protocol was approved by the hospitals directive board, we initiated data collection with a one week pilot before follow up. The pilot allowed the improvement of different aspects of the collecting form designed by the researchers. Children's parents were informed about the research objectives and a signed informed consent form was required to allow participation. 

A clinical nurse was instructed and trained in the detection of suspected ADRs in order to collect the data from all the patients that were admitted during the research period. A two sections instrument was used for data collection. The first section consisted of a questionnaire applied to children's parents, which included sociodemographic variables (gestational age, birth weight and height), personal and family medical history, information about medicines previously used and admission causes. The second section was an adaptation of The *Formato para Reporte de Sospecha de Eventos Adversos a Medicamentos* (FOREAM) made by the *Instituto de Vigilancia de Medicamentos y Alimentos* (INVIMA), which is similar to The Yellow Card Scheme. This form was used for collecting drug use data from nursing, medical and clinical laboratory tests records found on patients medical history.

We determined suspected ADRs cases according to the International Conference on Harmonisation (ICH), which defined a suspected ADR as "any deviation of the expected clinical status (signs, symptoms and other clinical and laboratory findings)" 12. When we suspected an ADR, we collected the information related to the onset and duration of symptoms, number of prescribed medicines to treat the ADR (e.g. antihistamines and corticosteroids), dose adjustment, treatment interruptions and patient outcome. In addition, all data associated with the detected changes was verified with pediatricians and nurses in charge.

Daily visits to the wards were conducted to identify new admissions, interview parents, detect suspected ADRs and take part on medical rounds. In other words, we developed an intensive drug monitoring plan from admission till patient discharge date, which was based on patient-centered intensive pharmacovigilance.

Once we finished gathering data from suspected ADRs, we employed Naranjo's algorithm to evaluate the temporal relationship and the biological/pharmacological plausibility between drug exposure and suspected ADR emergence. This let us establish imputability between drugs and suspected ADRs. Naranjos's algorithm classifies ADRs as doubtful (0), possible (1-4 points), probable (5-8 points) and definite (≥9) 13,14. Meanwhile, The severity of ADRs was assessed with the Modified Hartwig and Siegel Assessment Scale 15, which classifies ADRs as mild (uncomplicated primary disease which does not require treatment or the discontinuation of treatment), moderate (signs and symptoms appear but the functionality of organs and systems are not affected, but may require drug treatment) or severe (life-threatening symptoms or systemic organic dysfunction which require hospitalization or prolonged hospital stay and cause disability, persistent failure or defects, reduction of life expectancy or death). The Modified Schumock and Thornton's criteria 16 were used to establish ADRs preventability, in which an ADR was considered preventable when one or more of the questions defined in this tool, were affirmatively answered. The ADRs were classified according to the affected organ systems (integumentary, hematological, nervous, digestive, urinary, cardiovascular or respiratory). The multidisciplinary team employed for analyzing imputability, severity and preventability of suspected ADRs was formed by a pharmacist, a nurse, a pharmacologist and a pediatrician. The identified ADRs and the pharmacological groups related to them were classified according to the Task-force in Europe for Drug Development for the Young (TEDDY) 17 and The Anatomical Therapeutic Chemical Classification System (ATC) 18.

### Statistical analysis

The collected data was analyzed in the statistical program SPSS (Statistics Statistical Package for the Social Science, v21). ADRs incidence density was estimated. A descriptive analysis of the variables was conducted according to their nature. A crude bivariate relative risk (RR) and 95% confidence interval (CI) were calculated between the dependent variable, which was the presence or absence of ADRs, and different independent variables. A Chi-square test, with a significance level of *p* <0.05, was also performed between the dependent variable and each independent one.

### Ethical considerations 

The protocol was approved by the Research Ethics Committee of the Health Care Division of the "Universidad del Norte" and was declared as minimal risk by the researchers. Likewise, the protocol was conducted under the human research ethical criteria defined in the *Resolución 008430 de 1993* of the *Ministerio de Salud y Protección Social de Colombia* and The Declaration of Helsinki.

##  Results 

A total of 772 children were involved, of whom 49.1% were females. The average age was 12 months of age (range from 1 month to 60 months). Sixty point two percent of the children were <24 months of age and 46.6% of children were between 6 and 12 months of age. The most frequent medical diagnoses were: respiratory system diseases 40.7% (314); urinary tract diseases 22.4% (173); Skin and subcutaneous tissue disorders 19.2 % (148) and miscellaneous disorders 17.7% (137) (Table 1).

**Table 1.** Sociodemographic and clinical characteristics of participants.

Characteristics	Female N (%)	Total N (%)
Age (months)*		
<6	448 (5.7)	95 (12.3)
6-12	186 (24.1)	360 (46.6)
13-24	49 (6.3)	112 (14.5)
25-47	43 (5.6)	77 (10.0)
≥ 48	57 (7.4)	128 (16.6)
Medical diagnosis (ICD-10)		
Respiratory system diseases	146 (18.9)	314 (40.7)
Urinary tract diseases	79 (10.2)	173 (22.4)
Skin and subcutaneous tissue disorders	60 (7.8)	148 (19.2)
Miscellaneous	94 (12.2)	137 (17.7)
	**Mean (SD)**	**Mean (SD)**
Age, months: mean (SD)	20.3 (16.7)	20.8 (17.3)
Weight (Kg): mean (SD)	12.5 (6.9)	12.7 (7.1)
Length of hospitalization: Mean (SD)	5.5 (2.4)	5.6 (3.3)
Number of drugs by patient: mean (SD)	3.8 (2.0)	4.1 (2.5)

* Age range of all participants from 1 to 60 months. SD: standard deviation. ICD-10: International Classification of Diseases.

The average length of hospitalization was 5.6 ±3.3 days with a median of 5 days, and 72.2% had between 2 and 5 prescribed medicines (mean 4.1 ±2.5). 

### Incidence and characteristics of ADRs

A total of 156 ADRs were detected in 147 children, of which 138 children developed just one ADR during their hospitalization and 9 children presented two. The cumulative incidence of ADRs was 19.0% (147/772), with an incidence density of 37.6 ADRs every 1,000 patients -days (147/3,913). Furthermore, 98.1% (153) of the ADRs were classified as probable, 1.3% (2) possible and 0.6% (1) definite (certain). In addition 98.7% (154) were not preventable and 1.3% (2) preventable (these two were related to the rate of administration of vancomycin). In terms of severity, 66.0% (103) of the ADRs were mild and 34.0% (53) moderate (Table 2). The most affected organ systems were the digestive, cardiovascular and integumentary. The ADRs did not require treatment in 67.3% (105) of the cases and none of the patients showed sequels. In all cases, ADRs treatment was the responsibility of physicians.

**Table 2.** Frequency and characteristics of adverse drug reactions.

Characteristics		N (%)
Imputability (Naranjo`s Algorithm)	Definite (Certain)	1 (0.6)
	Probable	153 (98.1)
	Possible	23 (1.3)
Preventability (Schumock and Thornton criteria)	Preventable	2 (1.3)
	Not preventable	154 (98.7)
Severity (Modified Hartwig and Siegel assessment scale)	**Mild**	103 (66.0)
	Digestive: diarrhea (39), emesis (18), abdominal pain (3), loss of appetite (1)	61 (39.1)
	Cardiovascular: Tachycardia (21)	21 (13.5)
	Integumentary: rash (4), angioedema (2), erythema (2), urticaria (1), skin reaction (1)	10 (6.4)
	Site o application: phlebitis (6)	6 (3.8)
	Renal: Increased BUN (1), leg edema (1)	2 (1.3)
	Nervous: hyperactivity (1), somnolence (1)	2 (1.3)
	Hematologic: thrombocytopenia (1)	1 (0.6)
	**Moderate**	53 34.0)
	Digestive: diarrhea (30), emesis (6), abdominal pain (2), constipation (1)	39 (25.0)
	Integumentary: angioedema (2), erythema (2), urticaria (2), maculopapular erythema (1), rash (1), fever (1)	9 (5.9)
	Renal: Low urine output (1), genital edema (1)	2 (1.3)
	Site of application: phlebitis (1)	1 (0.6)
	Nervous: headache (1)	1 (0.6)
	Hematologic: Thrombocytopenia (1)	1 (0.6)

ADRs: Adverse Drugs Reactions

The therapeutic group that most frequently produced ADRs was systemic antibiotics 70.5% (110), in which ampicillin, amikacin and clarithromycin represented 43.6% (Table 3).

**Table 3.** Therapeutic groups related to ADRs.

ATC code (n=156 ADRs)		N (%)
Antibiotics		110 (70.5)
	Ampicillin	27 (17.3)
	Amikacin	26 (16.7)
	Clarithromycin	15 (9.6)
	Clindamycin	13 (8.3)
	Cephalothin	8 (5.1)
	Ceftriaxone	8 (5.1)
	Ampicillin + Sulbactam	4 (2.6)
	Others	9 (5.8)
Respiratory system		25 (16.0)
	Salbutamol	26 (16.0)
Systemic hormonal preparations		7 (4.5)
	Methylprednisolone	7 (4.5)
Nervous System		8 (5.1)
	Valproic acid	4 (2.6)
	Carbamazepine	1 (0.6)
	Diazepam	2 (1.3)
	Acetaminophen	1 (0.6)
Cardiovascular system		1 (0.6)
	Enalapril	1 (0.6)
Blood and blood forming organs		1 (0.6)
	Folic acid	1 (0.6)
Alimentary tract and metabolism		(2.7)
	Zinc sulfate	4 (2.7)

The Anatomical Therapeutic Chemical Classification System (ATC). ADRs: adverse drug reactions.

ADRs: adverse drug reactions.

### Factors associated with ADRs 

The mean age of children that developed ADRs was the same as the ones who did not showed any (Table 4). However, ADRs frequency was higher in children under 2 years of age (12.7%) than in children with two or more years of age (6.3%). 

**Table 4.** Comparison between Children patients with and without ADRs.

Variable	with ADRs (N= 147)*	without ADRs (N= 625)*	p
Age (months)	18.4 ± 15.8	21.3 ± 17.5	0.077
Length of hospitalization (days)	7.1 ± 5.2	5.3 ± 2.6	0.001
Number of prescribed drugs	5.0 ± 2.5	3.9 ± 2.4	0.001
Number of prescribed systemic antibiotics	2.0 ± 0.5	1.0 ± 0.5	0.001

ADRs: adverse drug reactions.

SD: standard deviation

*mean ± SD

The mean length of hospitalization in children who had ADRs was higher (7.1 days ± 5.2) than those who did not show ADRs (5.3 days ± 2.6, *p*= 0.001). The mean of prescribed medicines in children with ADRs was higher than those who did not showed any (mean 5.0 ± 2.5 vs 3.9 ± 2.4 drugs) (*p*= 0.001). Similarly, the number of prescribed systemic antibiotics in children with ADRs was also higher than in those who were not prescribed any (mean 2.0 ± 0.5 vs 1.0 ± 0.5) (*p*= 0.001) (Table 5). 

**Table 5.** Factors associated to ADRs.

Variables		n= 772	ADRs		RR	Chi	p value
			Yes (%)	No (%)	(95% CI)	Square		
Age (years)	2	465	12.7	47.5	1.32 (0.96-1.80)	3.13	0.07
	≥ 2	307	6.3	33.5			
Gender	Female	393	12.0	38.9	1.66 (1.22-2.25)	11.09	0.001
	Male	379	7.0	42.1			
Previous ADRs	Yes	42	1.5	3.9	1.54 (0.93-2.55)	2.61	0.106
	No	7306	17.5	77.1			
Systemic antibiotic	Yes	600	16.4	61.3	1.82 (1.17-2.82)	7.89	0.005
	No	1725	2.6	19.7			

ADRs: adverse drug reactions.

RR: relative risk.

IC: interval confidence.

ADRs= adverse drug reactions n=147

Male patients were more likely to develop ADRs (RR=1.32; 95% CI= 0.96-1.80, *p*= 0.001) than female patients. The use of systemic antibiotics was correlated with a higher risk of ADRs (RR= 1.82; (95% CI= 1.17-2.82, *p*= 0.005) than those who did not used an antibiotic (Table 5). 1.5% (12) of patients with ADRs reported previous ADRs.

## Discussion

This study encompasses an exhaustive collection and evaluation of ADRs in a cohort of 772 hospitalized pediatric patients. We identified an ADRs incidence of 19.0%, which is higher than the one found by Truner *et al*. 19, and Jimenez *et al*. 20, demonstrating that children are particularly susceptible to ADRs. The majority of ADRs found were not preventable, as in Temple *et al* 5. In order to prevent ADRs, it is advisable to generate strategies that are aimed at improving drug administration safety protocols.

Among the 147 children who presented ADRs, 138 children developed just one and 9 children develop two ADRs. It is noteworthy to mention that children with more than one ADR developed these manifestations at different times. Hence, by exhaustively evaluating patients showing more than one ADR, we concluded that second ADRs were not an extension of the previous ones. Similar data was found by dos Santos and Coelho 10, who reported that 25 patients developed just one, 5 presented two and 2 developed three ADRs. ADRs predisposition could be related to a prolonged exposure to more than one medication during hospitalization 10,21, and also to intrinsic biological factors typical of the pediatric age 22. In addition, we also found that younger children were more affected by ADRs than older ones. Analogous information was reported by Speranza *et al*. 23, and Aagaard *et al* 24. 

For its part, a cuban study which included pediatric patients under 18 years of age found that the age range most affected by ADRs was between two and eleven years of age 25. The difference between our results and the ones obtained in the previous study could be related to several things, such as patient's age, the defined age range and the number of patients included on it.

Our study only included children under 6 years of age divided in two age ranges (<2 years and equal or >2 years of age). This was made considering the remarkable biological variability and physiological changes that occur in this age range.

We also found that males were more often affected by ADRs than females. Comparable results were reported by The WHO ICSR database (VigiBase (r)), in which ADRs were predominantly present in males 9,10. Nevertheless, other studies have shown higher ADRs frequency in females 25,26.

The average length of hospitalization found was 7.1 days. Dos Santos and Coelho 10 reported an average length of 18 hospitalization days. This discrepancy might be related to differences in the pediatrics services provided by the facilities in both studies. Unlike dos Santos and Coelho study, the hospitals enrolled in our study did not have specialized pediatric services. Hence, it could be argued that children having more complex pathologies might need a longer time of hospitalization for the treatment of their condition. Still, other studies have reported similar results to ours 21. 

The average number of prescribed drugs in children with ADRs was similar to pediatric wards of European and non-European countries who have reported an average number higher than 5 27.

Systemic antibiotics and respiratory drugs were the therapeutic groups mostly associated with ADRs incidence. Antibiotic use was related to a higher risk of ADRs emergence, as reported by Oshikoyay *et al*. 28, and Martinez-Mir *et al*. 21, who found that antibiotics were the most common cause of ADRs. Other authors have also reported that ADRs are related with antibiotics use 10,21. As noted, antibiotics are not only the most prescribed class of drugs for hospitalized children, but also the ones that usually cause ADRs. We found that the most correlated ADRs associated with antibiotics were gastrointestinals. Therefore, the digestive system was the most affected by ADRs. This data is slightly consistent with evidence described by Sepahi *et al*. 29, who found that the integumentary, gastrointestinal and central nervous systems were the ones most commonly affected by ADRs. In addition, skin and gastrointestinal ADRs are the most diagnosed and reported events in pharmacovigilance 23,30. 

Most of the ADRs found in our work were mild. These results differ with the findings of Shamna *et al*. 31, who found that moderate ADRs were the most common. In our study, Naranjo's algorithm classified the majority of ADRs found as probable, while Vallejos 32 reported that the majority of ADRs were possible and none was definite. However, it is extremely difficult to compare various studies, since the estimation of ADRs incidence is greatly influenced by the definitions used, the methodology of detection and classification and the study setting. 

The main limitation of this study was the determination of imputability of adverse events. Despite patient daily monitoring, only 0.6% of ADRs found were labelled as definite. This was due to difficulties associated with case analysis by means of Naranjo's algorithm, since in order to accurately determine if an ADR is definite, drug readministration, placebo administration and drug serum levels lab tests must be carried out, which, in most cases, for ethical reasons, are not feasible. Moreover, in case of suspected ADRs, appropriate handling dictates that drugs must be suspended, which limits the ability to establish the remaining criteria for imputability determination. For these reasons, this study was purely observational and no intervention was made on patient's treatment by researchers. 

Although we did not calculate a sample size due to difficulties in establishing the general population, an observation period of six months allowed us to establish an ADR incidence which resulted in similar findings to those reported in other studies 10,21,24. 

In addition, the detection of suspected ADRs was performed by a trained nurse. Even though physicians are the main notifiers of ADRs in practice, clinical research and pediatric pharmacovigilance 24, it is clear that any health professional (physicians, nurses, pharmacists, dentists and others) can report suspected ADRs to pharmacovigilance systems. In addition, it should be noted that, due to the role nurses play in the administration and monitoring of therapy, they have a privileged position to detect drug effects, including ADRs 33. 

## Conclusions

This is the first study that monitors ADRs in general pediatric wards in Colombia. Therefore, it is an important contribution to drug safety in children. This study establish the 

ADRs incidence density, frequency and characteristics on a defined time frame for enrolled children under 6 years of age. This provides an initial approximation to drug safety in children. In addition, these results demonstrate that ADRs in hospitalized children are common and represent an additional burden of morbidity and risk for these patients. Thus, extra costs are placed in the health care systems, which is an aspect that should be studied in the future. 

The results obtained in this work are interesting not only for pharmacologist and pharmacoepidemiologist, but also for hospitals, medicine agencies, health care ministers, physicians, nurses and pediatricians. Hence, new programs should be developed to assess patient safety and the onset of adverse events during treatment and hospitalization. 

Hence, it is necessary to develop proactive pharmacovigilance and patient safety programs with a focus in risk analysis and management, in which ADRs reporting should be mandatory. This measure might help us make our health care systems safer, especially for children, in which this topic must be further investigated. 
